# Italian Children Exposure to Bisphenol A: Biomonitoring Data from the LIFE PERSUADED Project

**DOI:** 10.3390/ijerph182211846

**Published:** 2021-11-12

**Authors:** Sabrina Tait, Fabrizia Carli, Luca Busani, Demetrio Ciociaro, Veronica Della Latta, Annalisa Deodati, Enrica Fabbrizi, Anna Paola Pala, Francesca Maranghi, Roberta Tassinari, Giacomo Toffol, Stefano Cianfarani, Amalia Gastaldelli, Cinzia La Rocca, LIFE PERSUADED Project Group

**Affiliations:** 1 Center for Gender-Specific Medicine, Istituto Superiore di Sanità, Viale Regina Elena 299, 00161 Rome, Italy; sabrina.tait@iss.it (S.T.); luca.busani@iss.it (L.B.); francesca.maranghi@iss.it (F.M.); roberta.tassinari@iss.it (R.T.); 2 National Research Council, Institute of Clinical Physiology, Via Giuseppe Moruzzi 1, 56124 Pisa, Italy; fcarli@ifc.cnr.it (F.C.); ciociaro@ifc.cnr.it (D.C.); veronicadellalatta@hotmail.it (V.D.L.); pala@ifc.cnr.it (A.P.P.); amalia@ifc.cnr.it (A.G.); 3 Dipartimento Pediatrico, Universitario Ospedaliero “Bambino Gesù” Children’s Hospital, Piazza di Sant’Onofrio, 4, 00165 Rome, Italy; annalisa.deodati@opbg.net (A.D.); stefano.cianfarani@opbg.net (S.C.); 4 Unità Operativa Complessa Pediatria e Neonatologia, Ospedale Civile Augusto Murri, Via Augusto Murri, 21, 63900 Fermo, Italy; enrica.fabbrizi@libero.it; 5 Civitanova Marche Hospital, ASUR MARCHE Area Vasta 3, 62012 Civitanova Marche, Italy; 6 Associazione Culturale Pediatri, Via Montiferru, 6, 09070 Narbolia, Italy; giacomo@giacomotoffol.191.it; 7 Department of Systems Medicine, University of Rome Tor Vergata, Via Cracovia, 50, 00133 Rome, Italy; 8 Department of Women’s and Children’s Health, Karolinska Institutet and University Hospital, Solnavägen 1, 171 77 Stockholm, Sweden

**Keywords:** Bisphenol A, endocrine disruptor, children exposure, geographical areas, age, human biomonitoring

## Abstract

A human biomonitoring (HBM) study on bisphenol A (BPA) in Italian children and adolescents was performed within the LIFE PERSUADED project, considering the residing areas, sex and age. The median urinary BPA level was 7.02 µg/L, with children living in the South of Italy or in urban areas having higher levels than those residing in the North or in rural areas. Children aged 4–6 years had higher BPA levels than those aged 7–10 and 11–14 years, but no differences were detected between sexes. The exposure in Italian children was higher compared to children from other countries, but lower than the HBM guidance value (135 µg/L). The estimated daily intake was 0.17 μg/kg body weight (bw) per day, about 24-fold below the temporary Tolerable Daily Intake of 4 μg/kg bw per day established by the European Food Safety Authority. However, this threshold was exceeded in 1.44% of the enrolled children, raising concern about the overall exposure of Italian young population.

## 1. Introduction

Bisphenol A (BPA) is a widespread man-made chemical used in the production of polycarbonate plastics and epoxy-phenolic resins for food contact materials (microwave ovenware, food containers, refillable water containers, protective lining cans, etc.), and for non-food applications (paints, printing inks, carbonless and thermal paper, sealants used in dentistry, cosmetics, etc.). Chemical and physical conditions (e.g., pH, temperature) and prolonged use can promote the migration of BPA from manufacturing and processing facilities and packaging products into the environment and food, thus representing possible sources of human exposure [1].

BPA has been recognized as an endocrine disruptor (ED), initially for its estrogenic activity exerted via the estrogen receptors (ERα, ERβ and the estrogen-related receptor gamma, ERRγ). Recently, its mechanism of action as antagonist of the androgen (AR), thyroid (ThR) and peroxisome proliferator-activated gamma (PPARγ) receptors has been also reported [2,3,4].

Due to its endocrine-disrupting properties and the related toxicological effects on endocrine, reproductive, neurodevelopmental, immune and metabolic systems, in 2017 BPA was included in the Candidate List of substances of very high concern for human health by the European Chemical Agency (ECHA, https://echa.europa.eu/it/candidate-list-table/-/dislist/details/0b0236e180e22414, accessed on 20 October 2021). Moreover, the European Food Safety Authority (EFSA) re-evaluated the temporary Tolerable Daily Intake (t-TDI) for BPA, lowering the value to 4 μg/kg body weight (bw)/day [1].

Children’s exposure to BPA is of high relevance because their developmental life stage is particularly susceptible to ED effects, and health consequences can occur lifelong. Epidemiological studies suggest a role of BPA in the pathogenesis of several endocrine disorders in children including impairment of reproductive [5], behavioral and brain development [6], insulin resistance and obesity, and increased cardiometabolic risk [7,8].

To protect children, the European Commission prohibited the manufacture of baby bottles containing BPA in the European Union [9] and fixed legislation limits in toys and in food contact materials intended for infants or young children [10,11].

However, several human biomonitoring (HBM) studies revealed that children are still exposed to BPA [12,13,14]. Therefore, it is crucial to know the background exposure levels and set reference values in this vulnerable population group in order to perform a more appropriate risk assessment [15].

In this framework, the LIFE PERSUADED project has provided the missing HBM data on di-ethylhexyl phthalate (DEHP) and BPA exposure in the population of Italian children and their mothers. The detailed approach of the project, including the description of the HBM in mother–child pairs, the case-control studies on the association between exposure and precocious puberty or obesity in children and the in vivo studies on juvenile rats, was previously described [16].

In the HBM study, children of both sexes aged 4 to 14 years old were recruited in urban and rural areas of the North, Centre and South of Italy. The recently reported results on DEHP pointed out that children aged 4–6 years old and boys are the more exposed groups; moreover, exposure differences were observed among the different Italian geographic macro-areas [17].

The present paper reports the BPA background level of exposure in Italian children, and also estimates the BPA daily intake and Reference Values (RV95). All parameters were assessed considering the residing area (North, Centre and South of Italy, urban and rural areas), three age classes (4–6, 7–10 and 11–14 years old) and sex as factors influencing the exposure.

## 2. Materials and Methods

### 2.1. Study Population

The criteria of selection and enrollment of children in the LIFE PERSUADED project were described previously [16,17]. The whole study was approved by the Ethics Committee of the Istituto Superiore di Sanità. Briefly, a total of 900 healthy children of both sexes and across three age groups (4–6, 7–10 and 11–14 years old) were equally recruited; children were also identically distributed into the three geographical macro-areas (North, Centre, South), and in urban and rural residing areas of each macro-area, according to the criteria defined by the Italian Institute of Statistics (ISTAT, Rome, Italy, https://www.istat.it/, accessed on 20 October 2021). Thus, according to the stratum, the number of children was *N* = 300 for each macro-area, *N* = 450 for each residing area, *N* = 300 for each age group and *N* = 450 for each sex.

The enrolment of children was performed by family pediatricians of the Italian Health System (whose list is available on the project website, https://lifp.iss.it/?p=73 accessed on 20 October 2021), who joined the project through the Associazione Culturale Pediatri (ACP, https://acp.it/it/ accessed on 20 October 2021) and the Federazione Italiana Medici Pediatri Marche (FIMPM, http://www.fimpmarche.it/ accessed on 20 October 2021). Enrolment lasted from January 2015 to November 2017, after training meetings.

The following inclusion criteria were established: residence in the selected area for at least 6 months prior to the enrollment, healthy status, children body mass index between 5th and 85th percentile, only one child per family and age between 4 and 14 years old. Parents were asked to sign the consent form, and fill in a questionnaire on lifestyle and eating habits of their children, and a food diary on what the children ate during the two days before urine sampling. Data from questionnaires and food diaries will be presented in a future publication. At home, for each recruited child, a first morning urine sample was collected in a disposable container (BPA and phthalate-free) provided by pediatricians. The collection of the first morning urine is the suitable procedure adopted in HBM studies in children [18]. Pediatricians labeled each urine sample, questionnaire and food diary with a unique alphanumeric code, and stored the urine sample at −20 °C until delivery to the National Research Council (CNR) laboratory under controlled temperature.

### 2.2. Analytical Method

The urine samples were checked for their integrity upon arrival at the CNR laboratory, aliquoted in polypropylene (PP) tubes (BPA- and phthalate-free materials) for creatinine, BPA and DEHP metabolites determination (the latter was previously reported in [17]), and stored at −20 °C before being analyzed. Additional aliquots were stored in a biobank (https://www.ifc.cnr.it/index.php/it/biologia-preclinica/biobanca accessed on 20 October 2021).

Before analysis, urine samples were thawed at 4 °C and 500 μL of urine were collected in a glass tube and deconjugated with 2 μL of the β-glucuronidase enzyme (from Helix Pomatia enzyme aqueous solution, ≥100.000 units/mL; Sigma Aldrich, St Louis, MO, USA), 375 μL of ammonium acetate buffer (NH4CH3COO−) 1 M (Sigma Aldrich), at pH = 5 and 250 μL of MilliQ water (Merck Millipore, Burlington, MA, USA, “Milli-Q Synthesis A10). After overnight deconjugation at 37 °C, urine samples were extracted and purified using a C18-SPE column (SPE cartridge C18 ODS 3 mL tubes 200 mg, Agilent, Santa Clara, CA, USA). BPA was quantified by adding BPAd16 (CDN isotopes, Pointe-Claire, QC, Canada) as internal standard. Background levels, reagent blanks and spike recovery were measured in water and urine by adding BPAd16 as internal standard.

The samples were derivatized with 10 µL of BSTFA 1% TMCS and 50 µL of acetonitrile (Sigma Aldrich, Merck KGaA, Darmstadt, Germany) and BPA analysis was performed by Gas Chromatography/Mass Spectrometry (GCMS; 7890A coupled with 5975 Agilent, Santa Clara, CA, USA) equipped with a capillary column (DB-5MS J & W, l 30 m; i.d. 0.25 mm; film thickness 0.25 μm) in selected ion monitoring (SIM) mode by analyzing the fragment ions 357 for BPA and 368 for BPAd16.

The Limit of Detection (LOD) and Limit of Quantification (LOQ), calculated according to U.S. EPA procedure [19], were 0.157 ng/mL and 0.523 ng/mL, respectively.

Calibration curves and Quality Control samples were prepared by spiking known amounts of BPA and BPAd16, obtaining optimal linearity and accuracy (R^2^ = 0.9938 for the calibration curve). Sample concentrations were calculated from the calibration curve.

The analytical method was validated in a proficiency test organized by the HBM4EU Project within the Quality Assurance and Quality Control program, with good results. (https://www.hbm4eu.eu/online-library/?mdocs-cat=mdocs-cat-20&mdocs-att=null# accessed on 20 October 2021) [20].

BPA concentrations were normalized to urinary creatinine concentrations measured by Jaffe’s method (Beckman Coulter AU400, Brea, CA, USA) according to the manufacturer’s procedure. Creatinine levels were in the range 0.3–3 g/L in all urine samples included in the study [21]. BPA levels (as sum of free and glucuronide forms) are expressed both as unadjusted (μg/L) and creatinine-adjusted urinary levels (μg/g creatinine).

The overall data of BPA detected in the 900 children will be soon available on the Information Platform for Chemical Monitoring of the European Commission (IPCHEM, https://ipchem.jrc.ec.europa.eu/ accessed on 20 October 2021), as already undertaken for DEHP biomonitoring data.

### 2.3. Estimation of BPA Daily Intake

The BPA daily intake based on BPA creatinine-adjusted levels was calculated for each child, according to the formula applied by Covaci et al. [13]:

BPA Intake (μg/kg bw per day) = [*BPAcrea* (μg/g crea) × *crea per day* (g/day)]/bw (kg) (1)

where *BPAcrea* is the urinary concentration of BPA adjusted for creatinine, bw is the measured child body weight, *crea per day* is the estimated 24 h urinary creatinine excretion, calculated for each child as following:

*crea per day* = *creatinine conc* (g/L) × *daily urinary volume* (2)

where *creatinine conc* is the creatinine value measured for each urine sample. The *daily urinary volume* is calculated as (0.024 L × bw (kg)), considering a mean value of 1 mL/h/kg bw for children aged over 4 years old [22] and the body weight measured for each enrolled child.

The BPA daily intake was estimated for the total children population; moreover, to evaluate possible differences, comparison among children subgroups according to macro-area, living area, age and sex were performed. Furthermore, comparisons between sexes, residing area and among age classes in each macro-area were also performed.

### 2.4. Derivation of Reference Values

The methodology applied for the derivation of BPA Reference Values (95th percentile and 95% confidence intervals, RV95) was previously described [17]. In this study, RV95 values were calculated by excluding the subjects with a BPA unadjusted urinary level higher than 41.67 µg/L, leading to daily intakes ≥1 µg/kg bw per day; this value is conservative compared to the EFSA t-TDI of 4 μg/kg bw per day [1].

RV95 values were determined for the total population and for all the subgroups considered in the study, i.e., macro-areas, urban and rural areas, age and sex. Additionally, RV95 values were also calculated considering macro-area/area, macro-area/sex and macro-area/age subgroups.

### 2.5. Statistical Analysis

Statistical analysis was performed with STATA 14.2 (StataCorp, College Station, TX, USA) setting significance at *p* < 0.05 for all the statistical tests performed, as previously described [17]. Briefly, non-parametric Mann–Whitney and Kruskal–Wallis tests were used for comparisons among groups, with Dunn’s post hoc evaluation where applicable, for both unadjusted and creatinine-adjusted urinary levels.

According to EFSA, a left-censored limit of 0.262 μg/L (LOQ/2) was assigned to samples with a concentration below the LOQ of the method [23].

Spearman’s test with Bonferroni correction was used to calculate the correlation between data on BPA levels and the previously published sum of DEHP metabolites’ exposure levels in the same population of children [17].

## 3. Results

### 3.1. BPA Levels in the Population of Italian Children 

In the population of 900 children analyzed for BPA urinary levels, 96.10% showed values above the LOQ, with geometric means of 7.06 μg/L and 6.77 μg/g for unadjusted and creatinine-adjusted levels, respectively (Table 1).

Table 2 shows the levels of BPA in children stratified by residing macro-area, area, sex and age. Regarding the three macro-areas, significantly higher BPA unadjusted urinary levels were found in children residing in Southern Italy compared to those residing in the North; no significant difference was observed for creatinine-adjusted levels. Children residing in urban areas had significantly higher BPA levels compared to those residing in rural areas, both as unadjusted and creatinine-adjusted levels.

No significant difference in BPA levels was observed between girls and boys.

Moreover, no difference was observed among BPA unadjusted urinary levels in the three age classes, but the creatinine-adjusted concentrations were significantly different among the three groups, decreasing from children aged 4–6 years to 7–10 years to 11–14 years.

### 3.2. BPA Levels by Living Area, Sex and Age in Each Macro-Area

To better define possible different exposure patterns, BPA levels were compared by residing area, sex and age within each macro-area (North, Centre and South).

**Figure 1:**
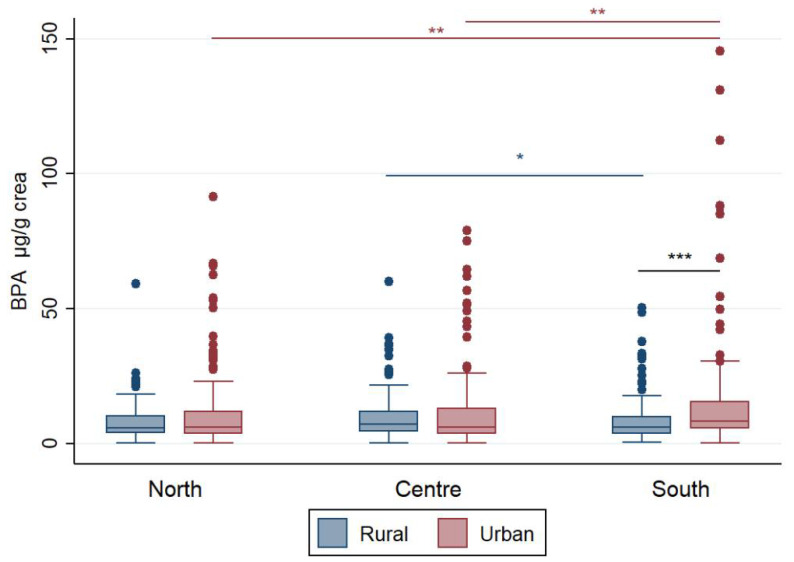
Box plots for BPA levels in urine samples of Italian children stratified by macro-area and area and normalized to urine creatinine content (µg/g). Outliers above 150 µg/g were excluded from the graph to improve box plot visibility. Asterisks indicate the level of significance: * *p* < 0.05, ** *p* < 0.01, *** *p* < 0.001.

Unadjusted and adjusted urinary BPA levels in children categorized by macro-area and area are summarized in Supplementary Table S1; further, creatinine-adjusted levels in these strata are shown in Figure 1.

Only in Southern Italy significantly higher BPA levels were found in children residing in urban areas compared to children living in rural areas, both as unadjusted and creatinine-adjusted concentrations. The exposure level of this group was also higher than that of children living in urban areas of Central Italy, for both types of urinary concentrations. Further, creatinine-adjusted urinary levels of Southern urban children were higher than those of Northern urban children.

Children residing in the rural areas of the Centre of Italy had higher unadjusted urinary levels compared to children living in rural areas of Northern Italy and higher creatinine-adjusted values compared to rural Southern children.

No significant differences were observed either between boys and girls in each of the three macro-areas or among boys and girls across the three macro-areas, as unadjusted or creatinine-adjusted values (Supplementary Table S2).

**Figure 2:**
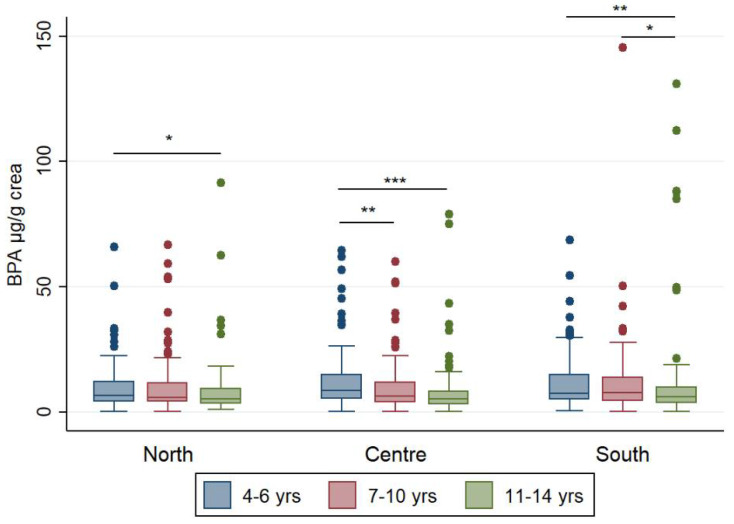
Box plots for BPA levels in urine samples of Italian children stratified by macro-area and age classes and normalized to urine creatinine content (µg/g). Outliers above 150 µg/g crea were excluded from the graph to improve box plot visibility. Asterisks indicate the level of significance: * *p* < 0.05, ** *p* < 0.01, *** *p* < 0.001.

BPA levels stratified by macro-area and age classes are summarized in Supplementary Table S3; creatinine-adjusted values are also displayed in Figure 2.

No differences were observed for unadjusted urinary BPA urinary levels across the three age classes in each macro-area, whereas BPA creatinine-adjusted concentrations in children aged 4–6 years were higher than those in children aged 11–14 years, in all macro-areas, and higher than those in children aged 7–10 years in Central Italy. Only in the South of Italy, children aged 7–10 years had higher BPA creatinine-adjusted levels than children aged 11–14 years.

### 3.3. Daily Intake Estimation

The geometric mean of the calculated daily intake of BPA for the total children population was 0.17 µg/kg bw per day (0.16–0.19 95% CI) (Table 3). Interestingly, 1.44% (*N* = 13) of the Italian children exceeded the t-TDI for BPA of 4 µg/kg bw per day [1] and 3.34% (*N* = 30) of the children had a daily intake between 1 and 4 µg/kg bw per day.

Statistical differences in daily intake values were observed only in relation to residing areas (Table 3). In particular, the daily intake in children of Southern Italy was higher than in the North, and was higher in urban than rural areas. As a confirmation, in the South, children living in the urban areas had higher daily intake than children in rural areas. Further, in the urban Southern macro-area, daily intake levels were higher compared to the other Italian urban areas. On the contrary, in rural Central Italy, children had higher daily intake levels compared to children living in the other two Italian rural areas.

### 3.4. Reference Values

The RV95 for BPA in the Italian children population (excluding the subjects with BPA exposure levels higher than 41.67 µg/L leading to daily intakes ≥1 µg/kg bw per day) was 24.06 µg/L (20.29–27.93, 95% CI). Overall, the RV95 for each category was close to the value for the total population (Table 4), but some groups deserve attention for having higher values than the upper bound of the general population, i.e., children aged 4–6 years, children from the South of Italy living in urban areas or aged 4–6 and 11–14 years, Southern girls, and children from Central Italy aged 4–6 years.

### 3.5. Correlation between BPA and Sum of DEHP Metabolites Levels

Urinary levels of BPA and of the sum of DEHP metabolites, reported in Tait et al. [17], were significantly correlated in the children population and in all the strata considered (Table 5), with the exception of children aged 11–14 yrs, for which the positive correlation was significant only for creatinine-adjusted concentrations.

## 4. Discussion

To the best of our knowledge, the LIFE PERSUADED project is the first HBM study evaluating BPA in Italian children (4–14 years old of both sexes), establishing background levels, BPA daily intakes and Reference Values by macro area, area, sex and classes of age.

As a main outcome, the LIFE PERSUADED HBM study demonstrated the widespread exposure to BPA in Italian children since measurable levels were found in 96.10% of the 900 enrolled subjects.

Notably, BPA levels in Italian children, both as a whole and within each age class, are about 3–4 fold higher than levels measured in children of similar age classes in other European countries, in USA and in Korea, but comparable to their 75th–95th percentile range levels (Table 6).

Potential differences in HBM data from different countries may be ascribed to methodological procedures and population characteristics, as previously highlighted [30]. The BPA analytical method was validated within the proficiency tests organized by the Quality Assurance Unit in the HBM4EU project [20]; thus, any possible bias due to analytical determination can be ignored. With respect to population characteristics, in LIFE PERSUADED, data were evaluated not only by residing area, but also according to sex and age categories, and taking into account body weight and creatinine levels, which are associated with a child’s age [22,31]. A general consensus on the best approach in HBM studies has not been reached yet; indeed, although creatinine-adjusted values are considered useful predictors of urinary BPA concentrations [32], they suffer some intra- and inter-individual variability in relation to sex and age, but also among different countries [28,30]. By assessing both volume-based and creatinine-adjusted BPA urinary levels, different levels of significance between groups have been highlighted, depending on the type of concentration considered, thus supporting the comparison of both.

Remarkably, BPA levels measured in children within LIFE PERSUADED are more similar to those measured in adults enrolled in two different areas in the Centre (3.59 µg/L, GM; 3.42–3.77 95% CI) and in the North of Italy (3.34 µg/L, GM; ± 1.69 Geometric SD) [33,34]. This evidence supports the hypothesis of a higher exposure in Italy, probably due to peculiar lifestyles, including, but not limited to, the use of plastics or food consumption habits. In this respect, the data analysis of questionnaires filled in by LIFE PERSUADED participants will clarify the main determinants of children’s exposure (manuscript in preparation). 

Anyway, the background levels in Italian children (7.06 µg/L, GM; 6.57–7.60 95% CI) are lower than the health-based HBM guidance values of 135 µg/L, derived for children population by the HBM4EU initiative, and thus do not indicate a risk for health from only BPA exposure [35].

The LIFE PERSUADED results on BPA exposure in Italian children showed significant differences when stratified by residing geographical area. Indeed, consistent with previous data on DEHP exposure in same children [17], BPA levels varied according to the residing macro-area but, differently from DEHP, were higher in Southern than in Northern Italy. These two Italian areas have distinct lifestyles and dietary habits which may drive the different exposure patterns; these factors are the subjects of another publication (manuscript in preparation). Nonetheless, we found that urinary levels of both plasticizers were positively correlated in the total population of children and in each macro-area, generally supporting similar exposures. 

Notably, BPA levels were also affected by the urbanization level: children living in urban areas had higher levels than those residing in rural areas (7.69 vs. 6.50 µg/L, GM), especially in the South of Italy, which also had higher levels compared to urban areas in Central Italy. With respect to rural areas, levels were higher in the Centre than in the South of Italy, thus representing a peculiarity in relation to the geographical pattern of BPA exposure. Previous reports from Denmark, Sweden and Spain did not show significant differences between BPA levels in rural and urban areas [36,37,38], possibly due to a lower population number or a consistent similarity between the territories. 

Sex seems not to have an impact on the exposure levels of BPA, even in the different geographical areas. On the contrary, age is a crucial determinant of exposure, because significantly higher concentrations were found in younger children compared to other age classes, and the pattern 4–6 > 7–10 > 11–14 was also generally confirmed within each macro-area. A previous report supports such evidence, showing higher BPA levels in youngest children compared to other classes of age [37]. Because the same exposure pattern was also observed in relation to DEHP exposure, the LIFE PERSUADED results indicated that younger children aged 4–6 years are the most exposed to the plasticizers BPA and DEHP in Italy [17].

Despite the higher internal levels found in Italian children in comparison with other studies, the estimated BPA daily intake in the same population was below the t-TDI of 4 μg/kg bw per day [1] in most of the subjects. Notably, 1.44% of the children exceeded this threshold, and 3.34% ranged from 1 to 4 μg/kg bw per day, and these outliers indicate a potential warning situation. Indeed, the estimated daily intake values are higher than those calculated in other studies of children inside and outside EU (Table 5), particularly in the Italian Southern urban area. Correlation analysis of the 1.44% subgroup did not highlight a concomitant occurrence of high/outlier DEHP levels in these children (data not shown), thus not providing evidence for a high exposure to plasticizers but for a specific BPA exposure condition.

The EFSA CEF Panel established the t-TDI value based on a BMDL (Benchmark Dose Lower Confidence Limit) of 8.960 mg/kg bw per day in adult mice [1]. The toxicological study on juvenile rats performed in the LIFE PERSUADED project used BPA dose levels derived from children exposure levels measured in the present HBM study, thus simulating real exposure conditions. The in vivo study estimated a BMDL of 0.05 mg/kg bw and 1.33 mg/kg bw per day for male and female juvenile rats, respectively [39], which are about 180- and 7-fold below the BMDL used by the EFSA CEF Panel. Thus, considering those values, a lower t-TDI value may be obtained and it is likely, in this scenario, that the daily intake values in Italian children will exceed it.

Although both daily intake and background levels were found to be lower than the guidance levels, the present data raise concern about children’s exposure to BPA in Italy. In this context, RV95 calculated according to age, sex and residing area may facilitate the identification of unusually high exposure levels, providing information for further monitoring studies or for mitigating actions [40]. Taking into account the function of RV95 in risk assessment, subjects with BPA daily intakes above 1 μg/kg per day were excluded because this value is too close to the t-TDI, therefore implying a risk. By excluding such values, we obtained a conservative RV95 of 24.06 µg/L for BPA in Italian children; however, some population groups exceeded the upper bound limit, such as children aged 4–6 years, the urban Southern children, and the Southern girls, confirming the concern for the higher exposure in this area. The Italian RV95 for children 4–14 years old is between the RV95 for children 3–5 and 6–14 years old (30 and 15 μg/L, respectively) derived by the German HBM Commission, despite the different exposure levels [41].

Overall, the LIFE PERSUADED results strongly support the need of data stratification according to geographical areas, age and sex to better differentiate exposure scenarios in risk assessment. More importantly, the results of this study raise concern about the potential impact of high BPA exposure levels on children’s health. Indeed, several studies, including LIFE PERSUADED, assessed the association of BPA exposure with obesity (unpublished data) or with behavioral problems in children, even at levels lower than those measured in Italy [42,43].

## 5. Conclusions

The LIFE PERSUADED biomonitoring study reinforces the evidence that younger children represent the population group with higher exposure to plasticizers, particularly in Italy. In this respect, measures to protect children’s health should be promoted by the Italian Health Authority in order to control and limit the sources of BPA exposure. In the framework of LIFE PERSUADED, several initiatives involving family pediatricians have been undertaken to inform the population about the risk of exposure to BPA and DEHP, and to promote the reduction of exposure through the adoption of different lifestyles. Among these measures, the most important was the publication and dissemination of the brochure “10 practical tips to limit the exposure to plasticizers in children and adults” (available on the website https://lifp.iss.it/ accessed on 20 October 2021).

The LIFE PERSUADED data on biomonitoring of children will contribute to BPA risk assessment in the EU re-evaluation process.

## Figures and Tables

**Table 1:** BPA levels in urine samples of Italian children (*N* = 900). The percentage of the samples > LOQ, the geometric mean (GM) with the 95% Confidence Interval (CI) and the median (P50) with the interquartile range (P25-75) are reported, for both unadjusted (µg/L) and creatinine-adjusted urinary concentrations (µg/g).

Units	>LOQ (%)	GM (95% CI)	P50 (P25–P75)
µg/L	96.10%	7.06 (6.57–7.60)	7.02 (4.16–12.06)
µg/g creatinine	96.10%	6.77 (6.27–7.30)	6.47 (3.92–12.77)

**Table 2:** BPA levels in urine samples of Italian children stratified by macro-areas, areas, sex and age. The geometric mean (GM) with the 95% CI and the median (P50) with the interquartile range (P25–P75) are shown for both unadjusted (µg/L) and creatinine-adjusted concentrations (µg/g).

	*N*	Units	GM (95% CI)	P50 (P25–P75)	*p*-Value
**Macro-Area**					
North ^a^	300	µg/L	7.14 (6.31–8.07)	6.69 (3.75–11.79)	
Centre	300		6.57 (5.76–7.50)	7.08 (4.40–11.15)	
South ^b^	300		7.53 (6.65–8.53)	7.84 (4.44–13.20)	0.0324 ^b vs. a^
North	300	µg/g crea	7.09 (6.26–8.04)	5.96 (3.64–12.10)	
Centre	300		6.29 (5.46–7.24)	6.62 (3.84–12.98)	
South	300		6.95 (6.11–7.90)	6.96 (4.22–12.59)	
**Area**					
Rural ^a^	450	µg/L	6.50 (6.10–7.03)	6.79 (4.10–10.71)	
Urban ^b^	450		7.69 (6.80–8.69)	7.34 (4.33–14.70)	0.0083 ^b vs. a^
Rural ^a^	450	µg/g crea	6.17 (5.68–6.72)	6.18 (3.83–10.63)	
Urban ^b^	450		7.42 (6.54–8.41)	7.28 (3.98–14.90)	0.0074 ^b vs. a^
**Sex**					
Boys	450	µg/L	7.28 (6.59–8.06)	7.34 (4.15–12.36)	
Girls	450		6.86 (6.17–7.63)	6.89 (4.18–11.63)	
Boys	450	µg/g crea	6.88 (6.20–7.64)	6.49 (3.86–12.10)	
Girls	450		6.66 (5.96–7.43)	6.41 (3.96–13.00)	
**Age**					
4–6 yrs old	300	µg/L	6.90 (6.10–7.83)	7.20 (4.30–11.95)	
7–11 yrs old	300		7.09 (6.25–8.04)	7.24 (4.14–11.63)	
11–14 yrs old	300		7.22 (6.34–8.22)	6.79 (4.11–12.57)	
4–6 yrs old ^a^	300	µg/g crea	7.95 (7.00–9.04)	7.90 (4.90–14.81)	<0.0001 ^a vs. c^
7–11 yrs old ^b^	300		6.85 (6.00–7.81)	6.74 (4.00–13.25)	0.0116 ^b vs. a^
11–14 yrs old ^c^	300		5.68 (4.97–6.50)	5.52 (3.27–9.65)	0.0031 ^c vs. b^

^a,b,c^ Superscript letters indicate groups with statistically significant differences (*p*-values < 0.05, e.g., ^a vs. b^).

**Table 3:** Estimated daily intake (µg/kg bw per day) of BPA in Italian children according to sex, age and residing area/macro-area expressed as geometric mean with 95% Confidence Intervals.

Category	Sub-Category (a)	Sub-Category (b)	Daily Intake GM (95% CI) (µg/kg bw per Day)	*p*-Value (a)	*p*-Value (b)
**Total**			0.17 (0.16–0.19)		
**Macro-areas**	North ^a^		0.17 (0.15–0.20)		
	Centre		0.16 (0.15–0.19)		
	South ^b^		0.18 (0.16–0.21)	0.0324 ^a vs. b^	
**Area**	Rural ^a^		0.16 (0.15–0.17)		
	Urban ^b^		0.19 (0.17–0.22)	0.0083 ^a vs. b^	
**Age class**	4–6 yrs		0.17 (0.15–0.19)		
	7–10 yrs		0.17 (0.16–0.20)		
	11–14 yrs		0.18 (0.16–0.20)		
**Sex**	Boys		0.18 (0.16–0.20)		
	Girls		0.17 (0.15–0.19)		
**Macro-area/Area**	North	Rural *	0.15 (0.13–0.17)	0.0492 ^N vs. C^	
		Urban *	0.20 (0.16–0.25)	0.0094 ^N vs. S^	
	Centre	Rural *	0.17 (0.15–0.20)		
		Urban *	0.16 (0.13–0.19)	0.0008 ^C vs. S^	
	South	Rural ^a,^*	0.15 (0.13–0.17)	0.0422 ^S vs. C^	
		Urban ^b,^*	0.23 (0.19–0.27)		0.0001 ^a vs. b^
**Macro-area/sex**	North	Boys	0.18 (0.15–0.21)		
		Girls	0.17 (0.14–0.20)		
	Centre	Boys	0.17 (0.14–0.20)		
		Girls	0.16 (0.14–0.19)		
	South	Boys	0.19 (0.16–0.22)		
		Girls	0.18 (0.15–0.21)		
**Macro-area/age**	North	4–6 yrs	0.16 (0.13–0.20)		
		7–10 yrs	0.19 (0.15–0.23)		
		11–14 yrs	0.18 (0.15–0.21)		
	Centre	4–6 yrs	0.18 (0.14–0.21)		
		7–10 yrs	0.15 (0.13–0.19)		
		11–14 yrs	0.17 (0.13–0.21)		
	South	4–6 yrs	0.18 (0.15–0.22)		
		7–10 yrs	0.19 (0.15–0.23)		
		11–14 yrs	0.19 (0.15–0.24)		

* Asterisks indicate significant differences among children living in urban or rural areas across the three macro-areas; ^a,b^; ^N,C,S^ Different superscript letters indicate statistically significant differences between groups; N = North; C = Centre; S = South.

**Table 4:** Reference values (RV95, P95 (95% CI)) for BPA in urine of Italian children (4–14 years old) according to sex and to the residing area/macro-area, excluding subjects with daily intake ≥1 µg//kg bw per day.

Category	Sub-Category (a)	Sub-Category (b)	*N*	RV95 (µg/L)
**Total**			855	24.06 (20.29–27.93)
**Macro-area**	North		284	22.82 (19.35–30.04)
	Centre		286	21.76 (17.16–31.48)
	South		285	27.88 (21.53–32.44)
**Area**	Rural		444	21.82 (17.56–28.56)
	Urban		411	25.74 (22.18–30.83)
**Age class**	4–6 yrs		290	29.07 (22.31–32.68)
	7–10 yrs		283	22.61 (18.51–29.01)
	11–14 yrs		282	23.71 (18.94–26.71)
**Sex**	Boys		428	23.56 (19.65–30.64)
	Girls		427	24.79 (20.91–28.74)
**Macro-area/Area**	North	Rural	150	19.31 (15.72–25.24)
		Urban	134	27.42 (21.88–33.06)
	Centre	Rural	147	23.59 (15.74–32.39)
		Urban	139	22.19 (17.80–34.05)
	South	Rural	147	27.89 (17.54–34.99)
		Urban	138	28.01 (20.63–32.87)
**Macro-area/sex**	North	Boys	143	22.26 (19.28–31.89)
		Girls	141	23.02 (17.84–30.78)
	Centre	Boys	144	25.76 (16.44–33.84)
		Girls	142	20.71 (16.00–32.49)
	South	Boys	141	24.32 (17.94–32.53)
		Girls	144	28.79 (22.52–35.12)
**Macro-area/age**	North	4–6 yrs	97	26.70 (22.10–32.81)
		7–10 yrs	93	25.32 (18.23–34.93)
		11–14 yrs	94	19.19 (16.80–24.99)
	Centre	4–6 yrs	97	31.22 (16.02–39.19)
		7–10 yrs	95	18.86 (14.00–32.83)
		11–14 yrs	94	26.13 (15.07–37.14)
	South	4–6 yrs	96	30.09 (19.73–36.68)
		7–10 yrs	95	27.67 (17.47–34.96)
		11–14 yrs	94	26.96 (19.72–37.75)

**Table 5:** Spearman correlations between BPA and sum of DEHP metabolite levels in urine samples of Italian children (*N* = 900) for both unadjusted (µg/L) and creatinine-adjusted urinary concentrations (µg/g) considering the different residing Italian macro-areas and areas, sex and age categories. Rho coefficients and *p*-values (Bonferroni corrected) are reported; significant p-values are in bold.

Category	Sub-Category	Unadjusted Concentrations		Creatinine-Adjusted Concentrations	
		Rho	*p*-Value	Rho	*p*-Value
**Total**		0.1524	**<0.0001**	0.2035	**<0.0001**
**Macro-area**	North	0.2149	**0.0002**	0.2234	**0.0001**
	Centre	0.1181	**0.0409**	0.2487	**<0.0001**
	South	0.1174	**0.0426**	0.1311	**0.0234**
**Area**	Rural	0.1662	**0.0004**	0.2386	**<0.0001**
	Urban	0.1408	**0.0028**	0.1719	**0.0003**
**Age class**	Boys	0.1023	**0.0302**	0.1457	**0.0020**
	Girls	0.1928	**<0.0001**	0.2627	**<0.0001**
	4–6 yrs	0.1865	**0.0012**	0.1373	**0.0174**
**Sex**	7–10 yrs	0.1823	**0.0015**	0.2112	**0.0002**
	11–14 yrs	0.0895	0.1225	0.1159	**0.0453**

**Table 6:** Overview of biomonitoring studies on BPA urinary levels in children expressed as geometric mean (GM) of unadjusted (μg/L) and creatinine-adjusted (μg/g crea) concentrations, 75th, 90th and 95th percentiles (μg/L) and estimated BPA daily intake (ng/kg bw per day).

Country	Collection (Years)	Population (*N*) (Age)	GM (μg/L) (μg/g crea)	P75	P90 (μg/L)	P95	Intake GM (95% CI) (ng/kg bw per Day)	References
Italy	2015–2017	900 4–14 years	7.06 μg/L 7.77 μg/g	12.1	22.98	24.1	170 (160–190)	This study
Germany	2014–2017	515 3–17 years	1.90 μg/L 1.67 μg/g	-	5.24	7.24	-	[24]
Slovenia	2011–2012	145 6–11 years	1.81 μg/L 1.51 μg/g	3.47	6.81	-	-	[25]
France	2014–2016	500 6–17 years	2.26 μg/L 1.97 μg/g	3.71	5.56	7.09	-	[26]
Portugal	2014–2015	110 4–18 years	1.58 μg/L 1.76 μg/g	-	-	16.0	41.6 (----)	[27]
Belgium	2011–2012	125 6–11 years	2.35 μg/L 2.10 μg/g	3.76	8.15	13.4	41.2 (32.9–51.7)	[13]
Denmark	2011–2012	142 6–11 years	1.87 μg/L 1.94 μg/g	3.42	6.20	7.90	38.9 (32.2–47.0)	[13]
Spain	2011–2012	118 6–11 years	1.83 μg/L 2.01 μg/g	3.59	7.36	9.84	38.3 (31.4–46.7)	[13]
Sweden	2011–2012	97 6–11 years	1.48 μg/L 1.67 μg/g	2.22	4.14	6.24	32.6 (27.8–38.3)	[13]
USA	2011–2012	396 8–10 years	1.6 μg/L 2.3 μg/g	3.1	-	8.5	30.4 (27.0–34.2)	[28]
Korea	2010–2012	258 7–12 years	1.88 μg/L 2.09 μg/g	-	-	-	40 (38–41)	[29]

## Supplementary Materials

The following are available online at https://www.mdpi.com/article/10.3390/ijerph182211846/s1, Table S1: BPA levels in urine samples of Italian children residing in rural or urban areas in the three macro-areas (*N* = 150). In the table are reported the geometric means (GM) with the 95% Confidence Interval (CI), the median (P50) and the interquartile range (P25–P75). Both unadjusted (μg/L) and creatinine-adjusted concentrations (μg/g) are reported, Table S2: BPA levels in urine samples of Italian boys and girls in each macro-area (*N* = 150). In the table are reported the geometric means (GM) with the 95% Confidence Interval (CI), the median (P50) and the interquartile range (P25–P75). Both unadjusted (μg/L) and creatinine-adjusted concentrations (μg/g) are reported, Table S3: BPA levels in urine samples of Italian children aged 4–6 years, 7–10 years and 11–14 years in each macro-area (*N* = 100). In the table are reported the geometric means (GM) with the 95% Confidence Interval (CI), the median (P50) and the interquartile range (P25–P75). Both unadjusted (μg/L) and creatinine-adjusted concentrations (μg/g) are reported.
