# Supplementary material for: Italian Children Exposure to Bisphenol A: Biomonitoring Data from the LIFE PERSUADED Project
Source: Int J Environ Res Public Health. 2021 Nov 12;18(22):11846. doi: 10.3390/ijerph182211846 (PMC8621164; doi:10.3390/ijerph182211846)
Supplement: Supplementary file 1 [file ijerph-18-11846-s001.zip › ijerph-1451859-supplementary.pdf]

Supplementary Table S1 - BPA levels in urine samples of Italian children residing in rural or urban areas in the three macro-areas (N=150). In the table are reported the geometric means (GM) with the 95% Confidence Interval (CI), the median (P50) and the interquartile range (P25-P75). Both unadjusted (µg/L) and creatinine-adjusted concentrations (µg/g) are reported.

Macro-Area (MA)	Area	Unit	GM (95% CI)	P50 (P25-P75)	p-value (area)	p-value (MA)
**North**	**Rural***	µg/L	6.20 (5.50-6.99)	6.62 (3.92-10.49)		
	**Urban**		8.21 (6.62-10.19)	6.69 (3.70-15.31)		
	**Rural***	µg/g crea	6.02 (5.32-6.81)	5.74 (3.73-10.10)		
	**Urban***		8.37 (6.74-10.39)	6.29 (3.63-17.32)		0.0094 [N vs. S]
**Centre**	**Rural***	µg/L	7.03 (6.06-8.16)	7.49 (4.86-10.71)		0.0492 [C vs. N]
	**Urban**		6.14 (4.94-7.64)	6.32 (3.93-12.38)		
	**Rural***	µg/g crea	6.78 (5.76-7.99)	7.18 (4.26-12.77)		0.0299 [C vs. S]
	**Urban**		5.83 (4.62-7.35)	6.09 (3.63-13.42)		
**South**	**Rural[a*]**	µg/L	6.30 (5.48-7.24)	6.28 (3.44-11.03)		0.0422 [S vs. C]
	**Urban[b*]**		9.00 (7.33-11.04)	9.22 (5.77-15.49)	0.0001[b vs. a]	0.0008 [S vs. C]
	**Rural[a*]**	µg/g crea	5.76 (4.97-6.69)	6.01 (3.60-9.80)		
	**Urban[b*]**		8.36 (6.80-10.29)	8.34 (5.39-15.77)	0.0002[b vs. a]	0.0032 [S vs. C]

[a,b] Asterisks indicate significant differences among children living in urban or rural areas across the three macro-areas; superscript letters beside p-values in the macro-area (MA) column (N=North, C=Center, S=South) indicate the significant pairwise comparison.

Supplementary Table S2 - BPA levels in urine samples of Italian boys and girls in each macro-area (N=150). In the table are reported the geometric means (GM) with the 95% Confidence Interval (CI), the median (P50) and the interquartile range (P25-P75). Both unadjusted (µg/L) and creatinine-adjusted concentrations (µg/g) are reported.

Macro-Area MA	Gender	Unit	GM (95% CI)	P50 (P25-P75)
**North**	**Boys**	µg/L	7.42 (6.26-8.80)	6.86 (3.85-12.93)
	**Girls**		6.86 (5.73-8.22)	6.40 (3.74-10.64)
	**Boys**	µg/g crea	7.42 (6.25-8.81)	6.34 (3.84-12.61)
	**Girls**		6.78 (5.64-8.15)	5.74 (3.50-11.33)
**Centre**	**Boys**	µg/L	6.59 (5.45-7.97)	7.25 (4.33-11.52)
	**Girls**		6.55 (5.45-7.88)	6.96 (4.41-10.86)
	**Boys**	µg/g crea	6.24 (5.09-7.64)	7.16 (3.77-11.86)
	**Girls**		6.34 (5.20-7.73)	6.38 (3.90-13.73)
**South**	**Boys**	µg/L	7.90 (6.70-9.32)	8.09 (4.41-12.89)
	**Girls**		7.18 (5.94-8.67)	7.29 (4.53-13.28)
	**Boys**	µg/g crea	7.04 (5.94-8.34)	6.68 (4.09-12.10)
	**Girls**		6.86 (5.64-8.34)	7.40 (4.37-13.08)

Supplementary Table S3 - BPA levels in urine samples of Italian children aged 4-6 years, 7-10 years and 11-14 years in each macro-area (N=100). In the table are reported the geometric means (GM) with the 95% Confidence Interval (CI), the median (P50) and the interquartile range (P25-P75). Both unadjusted (µg/L) and creatinine-adjusted concentrations (µg/g) are reported.

Macro-Area (MA)	Age	Unit	GM (95% CI)	P50 (P25-P75)	p-value (age)

**North**	**4-6 yrs**	µg/L	6.46 (5.13-8.15)	6.15 (3.79-10.90)	
	**7-10 yrs**		7.62 (6.07-9.56)	6.91 (3.84-12.74)	
	**11-14 yrs**		7.38 (6.13-8.88)	6.79 (3.75-11.62)	
	**4-6 yrs**[a]	µg/g crea	7.39 (5.87-9.29)	6.70 (3.97-12.79)	
	**7-10 yrs**		7.63 (6.05-9.61)	5.97 (4.10-13.62)	
	**11-14 yrs**[b]		6.33 (5.21-7.68)	5.27 (3.32-9.58)	0.0154 [b vs. a]
**Centre**	**4-6 yrs**	µg/L	7.05 (5.69-8.74)	7.64 (4.74-11.78)	
	**7-10 yrs**		6.13 (4.90-7.68)	7.16 (4.21-10.47)	
	**11-14 yrs**		6.57 (5.12-8.43)	6.57 (4.25-10.63)	
	**4-6 yrs**[a]	µg/g crea	8.44 (6.70-10.63)	8.58 (5.28-15.18)	
	**7-10 yrs**[b]		5.87 (4.62-7.47)	6.39 (3.75-11.84)	0.0054 [b vs. a]
	**11-14 yrs**[b]		5.02 (3.87-6.50)	5.25 (2.88-8.43)	<0.0001 [b vs. a]
**South**	**4-6 yrs**	µg/L	7.22 (5.84-8.92)	7.40 (4.70-13.17)	
	**7-10 yrs**		7.63 (6.23-9.36)	8.67 (4.44-12.62)	
	**11-14 yrs**		7.75 (6.10-9.85)	7.22 (4.31-13.87)	
	**4-6 yrs**[a]	µg/g crea	8.07 (6.53-9.98)	7.64 (4.91-14.98)	0.0062 [a vs. b]
	**7-10 yrs**[a]		7.17 (5.76-8.92)	7.68 (4.33-14.24)	0.0413 [a vs. b]
	**11-14 yrs**[b]		5.79 (4.56-7.36)	6.03 (3.64-10.00)	

[a,b] Superscript letters indicate significant differences among children of the three age classes across the three macro-areas.
